# LC-MS/MS quantification of sulfotransferases is better than conventional immunogenic methods in determining human liver SULT activities: implication in precision medicine

**DOI:** 10.1038/s41598-017-04202-w

**Published:** 2017-06-20

**Authors:** Cong Xie, Tong-meng Yan, Jia-mei Chen, Xiao-yan Li, Juan Zou, Li-jun Zhu, Lin-lin Lu, Ying Wang, Fu-yuan Zhou, Zhong-qiu Liu, Ming Hu

**Affiliations:** 1 Department of Pharmaceutics, School of Pharmaceutical Sciences, Southern Medical University, Guangzhou, Guangdong, 510515 China; 2 International Institute for Translational Chinese Medicine, Guangzhou University of Chinese Medicine, Guangzhou, Guangdong, 510006 China; 3 Department of Infectious Diseases, Nanfang Hospital, Southern Medical University, Guangzhou, Guangdong, 510515 China; 4 Department of Pharmacological and Pharmaceutical Sciences, College of Pharmacy, University of Houston, Houston, TX 77030 USA; 5 State Key Laboratory of Quality Research in Chinese Medicine, Macau University of Science and Technology, Macau (SAR), China

## Abstract

This study aims to determine whether enzyme activities are correlated with protein amounts and mRNA expression levels of five major human sulfotransferase (SULT) enzymes in 10 matched pericarcinomatous and hepatocellular carcinoma liver samples. The MRM UHPLC-MS/MS method, Western blot and RT-PCR were used along with SULT activity measurement using probe substrates. The LC-MS/MS method was specific for all five tested SULTs, whereas Western blot was specific for only two isoforms. The activities of SULT1A1, SULT1B1, SULT1E1 and SULT2A1 in 9 of 10 samples showed a significant decrease in tumor tissues relative to matched pericarcinomatous tissues, whereas the activities of SULT1A3 in 7 of 10 samples increased. The turnover numbers of SULTs did not change, except for SULT1A1. A generally high degree of correlations was observed between SULT activities and protein amounts (r^2^ ≥ 0.59 except one), whereas a low degree of correlations was observed between SULT activities and mRNA expression levels (r^2^ ≤ 0.48 except one). HCC reduced the SULT activities via impaired protein amounts. LC-MS/MS quantification of SULTs is highly reliable measurement of SULT activities, and may be adopted for implementing precision medicine with respect to drugs mainly metabolized by SULTs in healthy and HCC patients.

## Introduction

Precision medicine is a broad and rapidly advancing field in health care that considers the unique clinical, genetic, and environmental information of individuals^1^. Precision medicine was first practiced in oncology because of its potential to enhance targeted cancer therapies^2^. Precision medicine focuses on the following four significant aspects: human genetic information, transcription, protein and function/activity^3, 4^. Although defining genetic information and its transcription is quite common and easy, proteins and their activities should be determined to understand their biological in case of changes in their functions^5^. The latter may also enhance our understanding of the molecular pathogenesis of a disease^6^. Hence, proteins and their activities have the potential to serve as biomarkers associated with disease diagnosis, prognosis, stratification, and treatment^7^. Unfortunately, because of methodological shortcomings, protein amounts and activity are often not comprehensively evaluated, thereby hindering the implementation of precision medicine.

This scenario is particularly true for SULT enzymes, which catalyze sulfonation, a relatively understudied area in drug metabolism and clearance. In a typical human liver, four subfamilies of SULTs, namely, SULT1A, SULT1B, SULT1E, and SULT2A, are responsible for the metabolism of most marketed drugs^8^. SULT1A1 is generally recognized as the major xenobiotic-metabolizing SULT isoform that is responsible for the metabolism of reactive compounds (e.g., benzylic alcohols and aromatic hydroxylamines), which are associated with carcinogenesis^9^. Sulfate conjugation by other SULT enzymes (SULT1A3, SULT1E1 and SULT1B1) may result in the activation or inactivation of anti-cancer drugs (e.g., raloxifene, sesamol, fulvestrant, and resveratrol), thereby affecting their anti-tumor effects^10–13^. In addition, Huang LR *et al*.^14^ reported that SULT2A1 is involved in the phase II metabolism of dehydroepiandrosterone (DHEA) *in vivo* and the expression levels of dehydroepiandrosterone sulfotreansferase (DHEAS) are responsible for the difference in HCC. The expressions of SULT enzymes in tumors are crucial for cancer treatment because these proteins and enzymatic activities could be altered in the development of HCC^15^. Unfortunately, knowledge on the activities of SULT enzymes in HCC tumors remains limited. Moreover, only a few studies have directly assessed SULT protein expression and their functions, including specific enzyme kinetics and reaction rates in human tumor tissues. In comparison with phase I metabolism via important enzymes such as CYP3A4, SULTs do not always enjoy the abundance of reagents (e.g., lack of SULT isoform-specific and selective monoclonal antibodies or well characterized probe substrates) that are commonly associated with CYPs, the specific antibodies and substrates of which are often known and well characterized. In implementing precision medicine initiatives for SULT-mediated drug metabolism, the commonly used methods that will likely produce accurate results of enzyme activities must be identified. Identifying such methods is important because changes in key metabolism enzymes can lead to variability in the pharmacokinetic and pharmacodynamic properties of drugs (metabolized mainly by SULT enzymes) in patients and increase the possibility of therapeutic failure or severe toxicity. A comprehensive understanding of the influence of drug-metabolizing enzymes on drug disposition can assist the implementation of precision medicine^16^.

Currently, the established approach to explore SULT enzymes with respect to protein and mRNA expression levels in organs/tissues (S9) is focused on traditional bimolecular methods, such as Western blot and reverse-transcriptase polymerase chain reactions (RT-PCR)^17^. Western blot for SULTs lacks well-characterized SULT-specific and SULT-selective antibodies. Thus, its use is limited to semi-quantification, and it may fail to distinguish highly homologous proteins^8^. mRNA as the sole parameter to measure enzyme induction is prone to misinterpretation, which is often observed in overestimated induction potential^18^. Thus, traditional immunogenic methods do not meet the requirements of precision medicine.

MS-based quantifications are completely different from traditional immunogenic methods, such as Western blot analysis, which is generally restricted by the limited availability of specific antibodies. We can use the MRM UHPLC-MS/MS method to quantify the protein amounts of SULT enzymes. This method can improve the sensitivity (detecting peptides down to the femtomole range) and high throughput (simultaneously quantifying 50 or more proteins) of the absolute quantification of proteins, even those with high degrees of sequence homology (detecting peptides with varying molecular weights, which are less than 1 Da) in complex biologic matrices^19, 20^ or cultured cell lines^21^. Recently, we have developed an MRM UHPLC-MS/MS method to determine the absolute amounts of the isoforms of CYPs and UGTs in human liver tissues, and the results indicate the accuracy and reproducibility of this method and its potential to improve the understanding of hepatic disposition and drug metabolism^22^. In addition, this method can be used to distinguish different UGT1A isoforms, which are highly homologous proteins coded by a single gene with seven splicing variants.

In the present study, the activities, protein amounts and mRNA expression levels of the five major SULT enzymes were determined in 10 healthy liver samples, 10 HCC tumors and matched pericarcinomatous tissues. The differential expression of SULT enzymes in the tumor microenvironment was compared with those in pericarcinomatous tissues and healthy samples using the recently published LC-MS/MS method, which can simultaneously measure these SULT enzymes in one run even with a small sample size. Our data can demonstrate that LC-MS/MS effectively determines SULT enzymes levels and that it has the potential to aid the implementation of precision medicine in HCC patients who use drugs that are mainly metabolized by SULT enzymes.

## Methods

### Materials

Dopamine hydrochloride, 17β-estradiol 3-sulfate sodium salt, potassium 4-nitrophenyl sulfate, pregnenolone sulfate sodium salt, DHEA 3-sulfate sodium salt dehydrate, MgCl_2_, and 3′-phosphate 5′-phosphosulfate lithium salt hydrate (PAPS, >99% purity) were purchased from Sigma-Aldrich Co. (St. Louis, MO, USA). DHEA and 17β-estradiol were purchased from J&K Scientific LTD (Guangzhou, Guangdong, China). p-Nitrophenol and 2-aminophenol were obtained from Aladdin (Hang Zhou, Zhe Jiang, China). Dopamine 3-O-sulfate and dopamine 4-O-sulfate were purchased from TRC (Toronto, Ontario, Canada). The primary antibodies of SULT1A1, SULT1A3, SULT1B1, SULT1E1, and SULT2A1, as well as the GAPDH, were obtained from Santa Cruz Biotechnology (LA, California, USA) and Abcam (Cambridge, England, UK), respectively. TPCK (L-1-tosylamido-2-phenylethyl chloromethyl ketone)–treated trypsin was purchased from Promega (Madison, WI, USA). The recombinant human SULT isoforms (SULT1A1, SULT1A3, SULT1B1, SULT1E1 and SULT2A1) were purchased from R&D Systems (Toronto, Ontario, Canada). The solid phase extraction cartridges (C18 100 mg, 3 mL) were purchased from J.T. Baker (Philipsburg, NJ, USA). PrimeScript RT Reagent Kit and SYBR Premix Ex Taq II (TliRnaseH Plus) were purchased from Takara Bio (Shiga, Tokyo, Japan).

### Human liver samples

All investigations of human subjects were reviewed and approved by the NanFang Hospital of Southern Medical University Research Ethics Committee, Guangzhou, China. All experiments were carried out in accordance with the approved guidelines and regulations (Declaration of Helsinki). Informed consent for the use of all HCC patients for the experiments was obtained from all subjects. All the subjects had undergone anatomic or limited hepatectomy for HCC resection at the Affiliated NanFang Hospital of Southern Medical University. The HCC tissues and matched pericarcinomatous tissues (tissues that were macroscopically dissected from the tumor and were 1 cm away from tumor lesions) were obtained from 10 Chinese subjects (33–71 years old; 52 ± 11 years old, mean ± SD); the details are shown in Table 1. The surgical specimens were confirmed via pathologic examination and clinical pathological parameters. The HCC tissues were classified into five grades (grades cannot be assessed, are well-differentiated, are moderately differentiated, are poorly differentiated, and are undifferentiated) according to the AJCC Cancer Staging Manual^23^. Only the moderately and poorly differentiated cases were selected in the present study. The HCC and pericarcinomatous tissues were kept in ice-cold saline immediately after resection and then used in the preparation of liver S9 immediately within 30 min or stored in liquid nitrogen before RNA extraction.

**Table 1:** Human liver donor details and tissue histology.

HCC patients	Gender	Age(years)	Ethnicity	Histology and Histological Grade^a^	AFP (μg/L^)b^	Volume^c^ (cm^3^)	Healthy tissues	Gender^d^	Age (years)	Ethnicity
B03022	Male	59	Asian	HCC(M)	>1000	364.8	1	M	60	Caucasian
B03281	Male	58	Asian	HCC(M)	>1000	139.7	2	M	56	Caucasian
B04051	Male	39	Asian	HCC(M)	2.9	253.1	3	M	34	Caucasian
B05171	Male	71	Asian	HCC(P)	>1000	129.1	4	M	55	Caucasian
C07101	Male	42	Asian	HCC(P)	4834	499.4	5	M	44	Caucasian
C11051	Male	33	Asian	HCC(M)	3967	66.8	6	F	66	Caucasian
D01151	Male	53	Asian	HCC(M)	777	683.2	7	F	49	Caucasian
D05151	Male	53	Asian	HCC(M)	>1000	140.9	8	F	52	Caucasian
D10311	Male	56	Asian	HCC(M)	N/A	N/A	9	F	29	Caucasian
D12241	Male	51	Asian	HCC(M)	N/A	N/A	10	F	54	Caucasian

^a^HCC, Hepatocellular carcinoma; M, Moderately differentiated; P, Poorly differentiated.

^b^AFP, Alpha-fetoprotein.

^c^Tumor volume was determined by two-dimensional Volume (cm^3^) using the formula: length * width^2^/2, length and width of tumor were diagnosed by US or CT.

^d^M: male F: female.

### Preparation of human liver S9

The liver S9 fractions of the 10 HCC patients were processed with the protocol used previously with some minor modifications^21^. After washing with ice-cold buffer (8 mM KH_2_PO_4_, 5.6 mM Na_2_HPO_4_, 1.5 mM EDTA, 1 mM DTT, and 0.28 mM phenylmethylsulfoyl fluoride), the tissues were minced and homogenized in a solution containing 50 mM phosphate buffer (containing 250 mM sucrose and 1 mM EDTA, pH 7.4) and subsequently centrifuged at 9000 × *g* for 20 min at 4 °C. The obtained supernatant contained the S9 fractions and it was immediately stored at −80 °C^24^. The total protein concentration was measured using the Bradford method, with bovine serum albumin as the standard^25^. A set of healthy human liver S9 fractions (hHLS9-individual), which were prepared from 10 healthy livers without chronic diseases, was also obtained from BD Biosciences (Worburn, MA) according to similar procedures. The donor details and tissue histology are shown in Table 1.

### Total RNA extraction

The total RNA of the liver tissues was isolated and purified using a PureLink RNA Mini Kit purchased from Ambion, Life Technologies (Waltham, MA, USA) according to the manufacturer’s instructions. The concentration and purity of the total RNA were assessed using a Biospec-nano spectrophotometer (Shimadzu, Shiga, Tokyo, Japan). RNA quality was determined on the basis of A_260_/A_280_ ratio, which was 1.7 to 2.0 for all RNA preparations^26^.

### Absolute quantification of SULT enzymes in human liver S9 by using the MRM UHPLC-MS/MS method

The procedure is aimed at the selection of unique peptides for the MRM UHPLC-MS/MS method (Supplementary Fig. S1). We established the method for the absolute quantification of the SULT enzymes including SULT1A1, SULT1A3, SULT1B1, SULT1E1 and SULT2A1 in human SULT recombinant proteins, healthy human liver S9 samples (hHLS9-individual), HCC tumors (tHLS9-individual), and matched pericarcinomatous tissues (nHLS9-individual). The details of these methods were shown Supplementary Fig. S1 and Tables S1 and S2. The absolute expression amount of SULT1A1 was calculated indirectly by subtracting the SULT1A2 amount from the total amount of SULT1A1/1A2. A universal internal standard peptide (GYLPNPALQR) was designed for the quantification of these proteins. All the signature peptides were synthesized by APeptide Co. Ltd. (Shanghai, China), and their purity (>95%) was determined using HPLC-UV (with a detection wavelength of 220 nm) analysis and ESI-TOF-MS analysis. The stock solutions of all signature peptides were prepared in acetonitrile–water–formic acid (5: 95: 0.1, v/v/v).

The protein amounts of the five SULT enzymes were determined simultaneously through the MRM UHPLC-MS/MS method described previously, with some minor modifications^21^. The protein amounts were determined by quantifying proteotypic peptides produced through trypsin digestion^22^, using synthetic standard peptides (APeptode Co, Shanghai, China). All samples were analyzed using Agilent 1290 series UHPLC system and an Agilent 6490 Triple Quadruple mass spectrometer equipped with an electrospray ionization (ESI) source (Agilent Technologies). The LC column used for peptide separation was prepared using a Poroshell C18 column (2.1 mm × 100 mm, 2.7 μm) (Agilent Technologies) at 40 °C with a thermostated column oven. The mobile phase was composed of 0.1% formic acid water (A) and acetonitrile (B) using a gradient elution of 5–5% (v/v) B at 0–10 min, 5–45% B at 10–11 min, 45–80% B at 11–12 min, 80–5% B at 12–13 min. The flow-rate was 0.3 mL/min, and the injection volume was 5 μL. Quantification was performed in positive ion mode, and the mass spectrometer was configured to run a dynamic MRM experiment for peptides. The instrument settings were as follows: capillary voltage 3000 V; nebulizer gas, 30 psi; gas temperature, 15 °C; and sheath gas flow, 11 L/min. The MRM transitions are listed in Supplementary Table S2 and Supplementary Fig. S2. Generally, at least three MRM transitions are selected for protein quantification to obtain high sensitivity and high selectivity^22^. High sensitivity is achieved through quantitative ions. Selectivity is acquired by qualitative ions. Initially, we used the peptide standard to develop and optimize the MS method. After optimization, the highest response of two transitions was recorded for subsequent protein quantification. The reason is that in the process of tryptic digestion, the existence of matrix interference can affect quantitative ions in different degrees. Therefore, matrix interference may lead to the response of MRM pairs in peptide standard solutions and digested samples of liver tissues. Hence, we always selected the MRM pairs with the highest response in liver samples to detect protein amounts, which led to mostly y-ions, except for SULT1A3, in which the b-ions were better (Supplementary Fig. S3).

Calibration curves were established by plotting the peak area ratios of each selected MRM versus the concentrations of signature peptides that spiked. A weighting factor 1/x was applied. All calibration curves were required to have a correlation value of at least 0.99 (Supplementary Fig. S4).

### Western blot analyses

The healthy human liver S9 samples and the recombinant human SULT proteins were analyzed by using Western blot. The protein samples were loaded onto each lane and separated by SDS-PAGE (4% stacking gel, 10% separating gel). GAPDH served as the loading control. The procedures were performed according to the literature^27^.

### Measurement of SULT enzymatic activities in healthy, tumor and pericarcinomatous tissues

SULT1A1, SULT1A3, SULT1B1, SULT1E1, and SULT2A1 activities were measured using probe substrates. The sulfonation processes were observed in tHLS9-pooled and nHLS9-pooled collected from 10 HCC patients, and rHLS9-pooled was used as the reference. The assay details are provided in Supplementary Table S3. Assay conditions, such as protein concentration and incubation time, were validated to derive the linear enzyme kinetics. In general, a typical incubation mixture (final volume = 200 μL) containing 50 mM potassium phosphate buffer at pH 7.4 is used. Liver S9 (final concentration = 0.25 mg/mL) for measuring the activities of every probe substrate, except SULT1A3 (with a concentration of 0.5 mg/mL), was mixed with MgCl_2_ (1 mM) and PAPS (0.025 mM) prior to the addition of the substrates at varying concentrations (details are presented in Supplementary Table S4). The formation of metabolites of p-nitrophenol (SULT1A1), dopamine (SULT1A3), 2-aminophenol (SULT1B1), 17β-estradiol (SULT1E1), and DHEA (SULT2A1) were monitored using an Agilent 1290 Infinite UHPLC system coupled with Agilent G6490 triple quadruple mass spectrometer equipped with an ESI source in negative ionization mode. All the analytical methods were validated. The optimal instrument-dependent and compound-dependent parameters for measuring various SULT metabolites are summarized in Supplementary Table S4. MassHunter Acquisition Software Rev B6.00 (Agilent Technologies) was used for data acquisition.

### Real-time PCR Gene Expression Analysis

The full-length sequences of the target genes were obtained from GeneBank, and the primers (forward and reverse) were designed using Primer Premier 6.0 (Premier Biosoft, CA) software and according to the criteria described previously^28^. The uniqueness of the primer sequences was assessed by performing a BLAST search (National Center for Biotechnology Information). The details of the primers and inventory used in the present study are provided in Supplementary Table S5. All the oligonucleotide primers were custom-synthesized by BGI Technologies. (Shenzhen, China).

For first-strand cDNA synthesis, 1,600 ng of total RNA was reverse-transcribed to a final volume of 40 μL following the protocol for the PrimeScript^TM^ RT Reagent Kit. The reactions were diluted with water 20 times in all real-time PCR experiments. Real-time PCR was performed in an ABI 7500 Fast Real-Time PCR System (Applied Biosystems) using SYPR Premix Ex Taq^TM^ II. The PCR amplification had 40 cycles (95 °C for 5 s and 60 °C for 34 s) after an initial denaturation step (95 °C for 30 s). All measurements were performed in triplicate. The relative expression differences were calculated using the comparative ΔΔCt method^29^, and Ct values were normalized to GAPDH expression levels.

### Statistical analysis

The Shapiro–Wilk test of normality was performed in SPSS Statistics 17.0 software to check the distribution shape of the data. A paired-sample t-test was performed to analyze the normally distributed data. For non-normally distributed data, a Wilcoxon sign rank test was used. Correlation analyses were performed using a Pearson product-moment correlation for normally distributed data and Spearman rank correlation for non-normally distributed data. A *P* value of <0.05 was considered as the minimum level of statistical significance (two-tailed) for all the statistical analyses. The kinetic parameters were estimated to fit the proper models (Michaelis–Menten, autoactivation, substrate inhibition or biphasic kinetic) to the substrate concentrations and initial rates using GraphPad Prism 5.0 software, aided by profiles of the EadieHofstee plots as previously described^30^.

The calculation of the absolute amount of proteins used for MRM UHPLC-MS/MS was described previously^22^ and used with some minor modifications. The protein amounts of SULT enzymes can be calculated as follows:

1 $${{\bf{A}}}_{{\bf{SULT}}}=\frac{{{\bf{C}}}_{{\bf{peptide}}}\times {\bf{V}}}{{\bf{Amount}}\,{\bf{of}}\,{\bf{S9}}\,{\bf{protein}}}$$

where A_SULT_ (pmol/mg S9 protein) denontes the protein amounts of SULT enzymes in the human liver S9, C_peptide_ (pmol/μL) is the peptide concentration, and V is the complex volume (200 μL) before the UHPLC-MS/MS analysis; the amount of total S9 protein is 0.1 mg.

The turnover number (TON) indicates the catalytic capacity of enzymes. It is defined as the amount of substrate molecules that can be converted by an enzyme protein into a product per unit of time (a turnover rate). The TON can be calculated as follows:

2 $${\bf{TON}}=\frac{{\bf{V}}}{{\bf{Et}}}$$

where V is the reaction rate at a specific substrate concentration, and Et is the total enzyme concentration. As the protein amount of each SULT enzyme was quantified in hHLS9-individual, tHLS9-individual, and nHLS9-individual through the LC/MS-MS method, the TONs of the probe substrates metabolized by the SULT enzymes in the healthy, pericarcinomatous, and tumor tissues were obtained.

## Results

### Comparison between Western blot, and the MRM UHPLC-MS/MS method with regard to analysis specificity and speed of analysis

**Figure 1:**
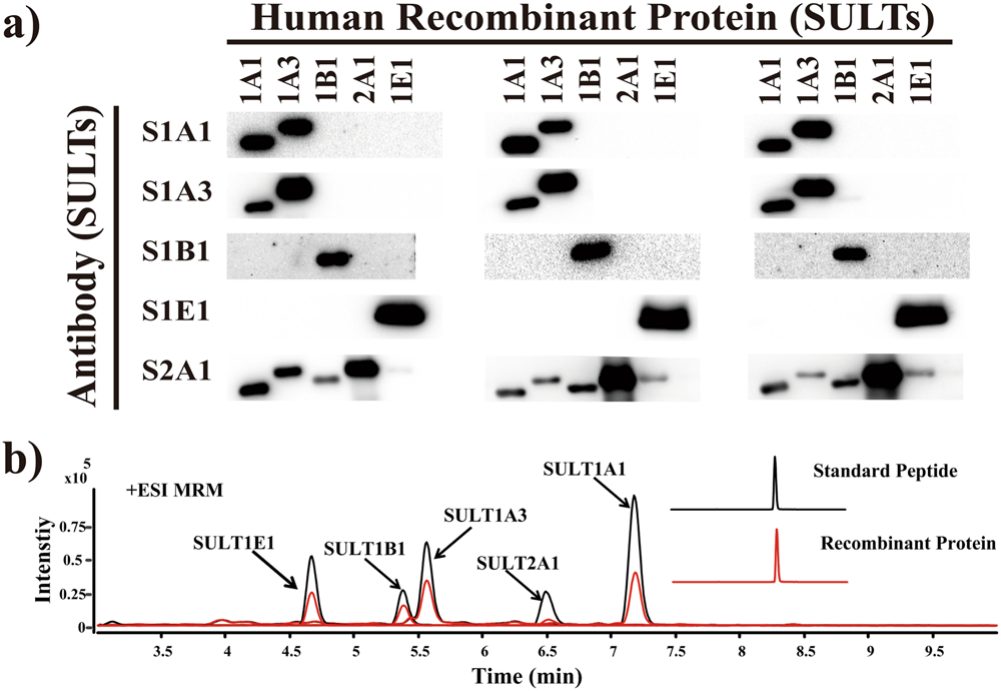
(**a**) SULT enzyme antibodies were used to analyze human recombinant proteins including SULT1A1, SULT1A3, SULT1B1, SULT1E1 and SULT2A1. The same experiments were repeated three times. (**b**) The black peak represents the MRM chromatograms of the tryptic peptides derived from healthy human liver S9 samples, and the chromatograms of the five tryptic peptides were detected through quantitative MRM transitions. The red peak represents the MRM chromatograms of SULT human recombinant proteins.

To validate the specificity of the analysis method, we used the Western blot analysis and MRM UHPLC-MS/MS methods in analyzing human recombinant SULT proteins, namely, SULT1A1, SULT1A3, SULT1B1, SULT1E1 and SULT2A1. As shown in Fig. 1(a), only 40% of the antibodies displayed specificities. As shown in Fig. 1(b), 100% of the MRM UHPLC-MS/MS displayed absolute specificity. The Western blot analysis took 24 h, whereas the MRM UHPLC-MS/MS method finished the analysis in less than 10 min, although sample preparation took about 6 h^31, 32^.

### SULT enzyme protein quantification and correlation plots in 10 healthy human liver S9 samples

**Figure 2:**
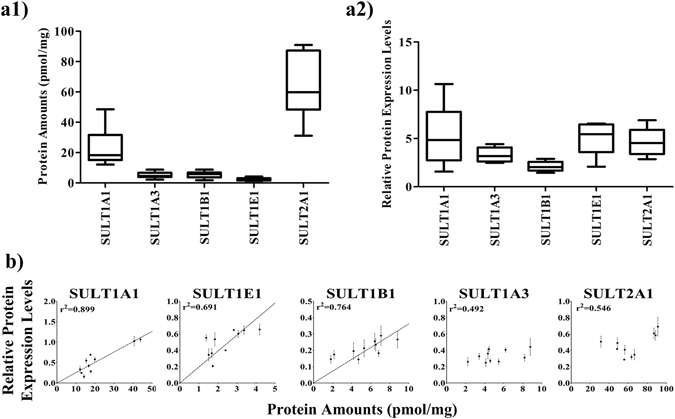
(**a1**,**a2**) Boxplots show the average protein amounts and relative protein expression levels of SULT1A1, SULT1A3, SULT1B1, SULT1E1, and SULT2A1 in the liver S9 samples from 10 healthy human donors, as determined by the MRM UHPLC-MS/MS method and Western blot analysis. (**b**) Correlation between the protein amounts and relative protein expression levels of SULT enzymes in hHLS9-individual. (SULT1A1, r^2^ = 0.899; SULT1B1, r^2^ = 0.764; SULT1E1, r^2^ = 0.691; SULT1A3, r^2^ = 0.492; SULT2A1, r^2^ = 0.546).

We used MRM UHPLC-MS/MS to quantify five SULT enzymes, namely, SULT1A1, SULT1A3, SULT1B1, SULT1E1, and SULT2A1, in 10 healthy human liver S9 samples (Fig. 2(a1) and Supplementary Table S6). The protein amounts of SULT2A1 and SULT1A1 were 63.6 and 23.7 pmol/mg, respectively; these amounts appeared to be much higher than those of the other three enzymes. The protein levels of these five SULT enzymes were also determined using Western blot (Supplementary Fig. S3). As shown in Fig. 2(a2) and Supplementary Table S6, the Western blot analysis revealed relatively small differences among the five SULT isoforms. We then plotted the absolute protein amounts (from LC-MS/MS) and relative protein expression levels (from Western blot) (Fig. 2(b) and see Supplementary Table S6). As expected, the plots of SULT1B1 and SULT1E1 showed excellent correlation coefficients because the antibodies were specific. By contrast, SULT1A3 and SULT2A1 showed poor correlation because the antibodies displayed poor specificities (r^2^ = 0.492, r^2^ = 0.546). SULT1A1, which was expressed at significantly high levels in our LC-MS/MS measurement, displayed good correlation because its expression levels were much higher than those of the interfering SULT1A3 in human liver S9 (Supplementary Fig. S3), thereby suppressing the impact of SULT1A3 interference. The present LC-MS/MS method was validated in terms of intra- and inter-day precision and accuracy, recovery, and matrix effect (Supplementary Tables S7, S8 and S9).

### Change in kinetics profiles of SULT enzymes in tumor tissues of HCC patients

The metabolic activities and catalytic functions of SULT enzymes in HCC are rarely addressed in China. Thus, using probe substrates, we systematically measured the metabolic functions of SULT1A1, SULT1A3, SULT1B1, SULT1E1, and SULT2A1 in tumor S9 (tHLS9-pooled), pericarcinomatous S9 (nHLS9-pooled), and reference pooled normal human S9 (rHLS9-pooled). As a rare exception, the apparent kinetic mechanism was the same for all the pooled samples, except for SULT2A1, in which rHLS9-pooled displayed a mechanism (Michaelis–Menten) different from those of the other two types of S9 (substrate inhibition) (Table 2).

**Table 2:** Summarize of enzyme kinetic parameters for five SULT activities in pooled human Liver S9.

Isoforms	Kinetic Parameters	nHLS9s-pooled	tHLS9s-pooled	rHLS9-pooled
SULT1A1^a^	Km (μM)	0.88 ± 0.20	0.82 ± 0.06	2.63 ± 0.16
	V_max_ (pmol/mg/min)	2004.77 ± 102.28	451.31 ± 6.88	3679.89 ± 67.25
4-Nitrophenyl sulfate	CL (V_max_/K_m_, μl/mg/min)	2285.46	550.15	1400.78
	Kinetic mechanism	M-M^b^	M-M^b^	M-M^b^
SULT1A3	K_m_ (μM)	0.17 ± 0.06	0.83 ± 0.22	0.41 ± 0.09
	V_max_ (pmol/mg/min)	35.27 ± 2.52	101.66 ± 10.07	38.67 ± 2.32
Dopamine 3-o-sulfate^#1^	CL (V_max_/K_m_, μl/mg/min)	201.77	122.12	95.15
	Kinetic mechanism	Substrate inhibition	Substrate inhibition	Substrate inhibition
	K_m_ (μM)	0.53 ± 0.18	0.95 ± 0.14	0.76 ± 0.19
	V_max_ (pmol/mg/min)	6.53 ± 0.88	17.88 ± 0.97	6.66 ± 0.56
Dopamine 4-o-sulfate^#2^	CL (V_max_/K_m_, μl/mg/min)	12.29	18.88	8.75
	Kinetic mechanism	Substrate inhibition	Substrate inhibition	Substrate inhibition
SULT1B1	K_m_ (μM)	2.78 ± 0.19	2.38 ± 0.36	13.18 ± 2.50
	V_max_ (pmol/mg/min)	14.07 ± 0.22	6.12 ± 0.21	14.38 ± 1.20
Aminphenol-sulfate	CL (V_max_/K_m_, μl/mg/min)	5.07	2.57	1.09
	Kinetic mechanism	M-M^b^	M-M^b^	M-M^b^
SULT1E1	K_m_ (μM)	10.85 ± 1.20	9.33 ± 0.92	8.11 ± 1.63
	V_max_ (pmol/mg/min)	76.52 ± 3.56	30.21 ± 1.19	44.69 ± 5.30
β-Estradiol 3-sulfate	CL (V_max_/K_m_, μl/mg/min)	7.05	3.24	5.51
	Kinetic mechanism	Biphasic	Biphasic	Biphasic
SULT2A1	K_m_ (μM)	0.73 ± 0.15	0.70 ± 0.12	0.81 ± 0.08
	V_max_ (pmol/mg/min)	95.14 ± 7.68	45.46 ± 3.44	81.98 ± 1.65
DHEA 3-sulfate	CL (V_max_/K_m_, μl/mg/min)	129.57	64.56	101.26
	Kinetic mechanism	Substrate inhibition	Substrate inhibition	M-M^b^

^a^Probe substrate and its metabolite.

^b^M-M, Michaelis-Menten.

The isomers #1(Dopamine 3-o-sulfate) and #2(Dopamine 4-o-sulfate) are the two sulfation metabolites.

**Figure 3:**
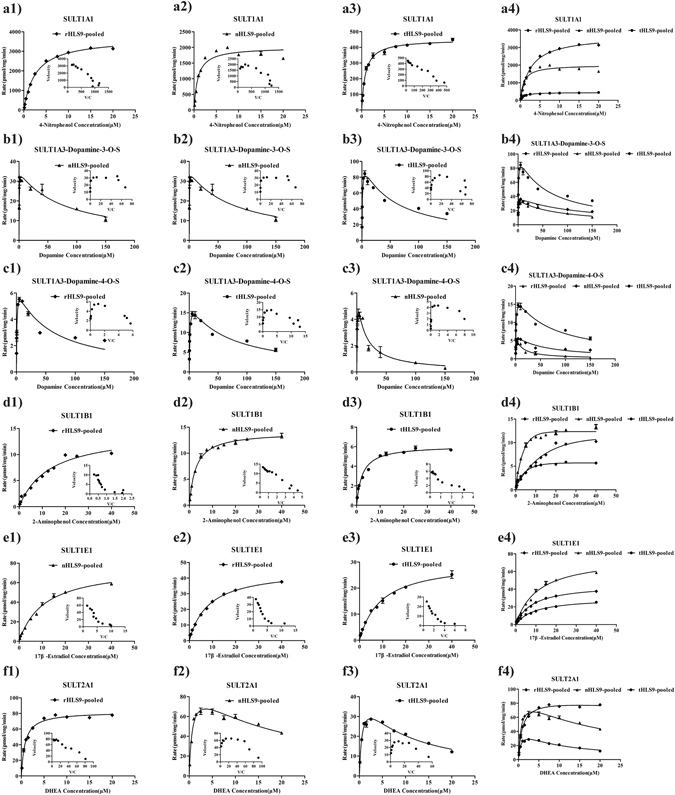
Enzyme kinetic analysis for the measurement of the activities of the five SULTs in rHLS9-pooled ((**a1**–**f1**), diamonds), nHLS9-pooled ((**a2**–**f2**), triangles), and tHLS9-pooled ((**a3**–**f3**), circles) through specific probe substrates. In each figure, the inset shows the Eadie-Hofstee plot. (**a4**–**f4**) show the comparison among the activities of the SULT isoforms in rHLS9-, nHLS9-, and tHLS9-pooled. Each data point represents an average of three determinations, and the error bar represents the SD of the mean (n = 3).

The V_max_ values derived using different pooled S9 preparations were highly variable when using different probe substrates. As shown in Fig. 3 and Table 2, in terms of SULT1A1 activities, the V_max_ value of nHLS9-pooled was smaller than those of rHLS9-pooled (V_max_: 2004.77 ± 102.28 pmol/mg/min vs. 3679.89 ± 67.25 pmol/mg/min); both V_max_ values were much higher than that of tHLS9-pooled (451.3 ± 6.88 pmol/mg/min). In terms of SULT1A3, the V_max_ values of nHLS9-pooled were the same as those of rHLS9-pooled (V_max_: 35.27 ± 2.52 pmol/mg/min vs. 38.67 ± 2.32 pmol/mg/min); both values were much lower than those of tHLS9-pooled (101.7 ± 10.1 pmol/mg/min). In terms of SULT1B1, the V_max_ values of nHLS9-pooled were the same as those of rHLS9-pooled (V_max_: 14.07 ± 0.22 pmol/mg/min vs. 14.38 ± 1.20 pmol/mg/min); both values were higher than those of tHLS9-pooled (6.128 ± 0.21 pmol/mg/min). In terms of SULT1E1, the V_max_ values of nHLS9-pooled were higher than those of rHLS9-pooled (V_max_: 76.52 ± 3.56 pmol/mg/min vs. 44.69 ± 5.30 pmol/mg/min), both values were higher than those of tHLS9-pooled (30.21 ± 1.19 pmol/mg/min).In terms of SULT2A1, the V_max_ values of nHLS9-pooled were comparable to those of rHLS9-pooled (V_max_: 95.14 ± 7.68 pmol/mg/min vs. 81.98 ± 1.65 pmol/mg/min); both values were higher than those of tHLS9-pooled (45.46 ± 3.44 pmol/mg/min) (Table 2).

Intrinsic clearance values (V_max_/K_m_) is sometimes useful in determining *in vivo* clearance. Hence, a short summary of the differences in trend was made and presented. For SULT1A1, the trend was nHLS9-pooled > rHLS9-pooled > tHLS9-pooled (≈4:3:1). For SULT1A3, the trend was nHLS9-pooled > tHLS9-pooled > rHLS9-pooled (≈2:1:1). For SULT1B1, the trend was nHLS9-pooled > tHLS9-pooled > rHLS9-pooled (≈5:2:1). For SULT1E1, the trend was nHLS9-pooled > tHLS9-pooled > rHLS9-pooled (≈7:5:3). For SULT2A1, the trend was nHLS9-pooled > rHLS9-pooled > tHLS9-pooled (≈2:2:1).

For K_m_ values, we also observed large differences in certain samples but not in others. However, we will not include the details here about their variability. Interested readers could refer to the values in Table 2.

### Decrease in SULT enzymatic activities in tumor tissues of HCC patients

**Figure 4:**
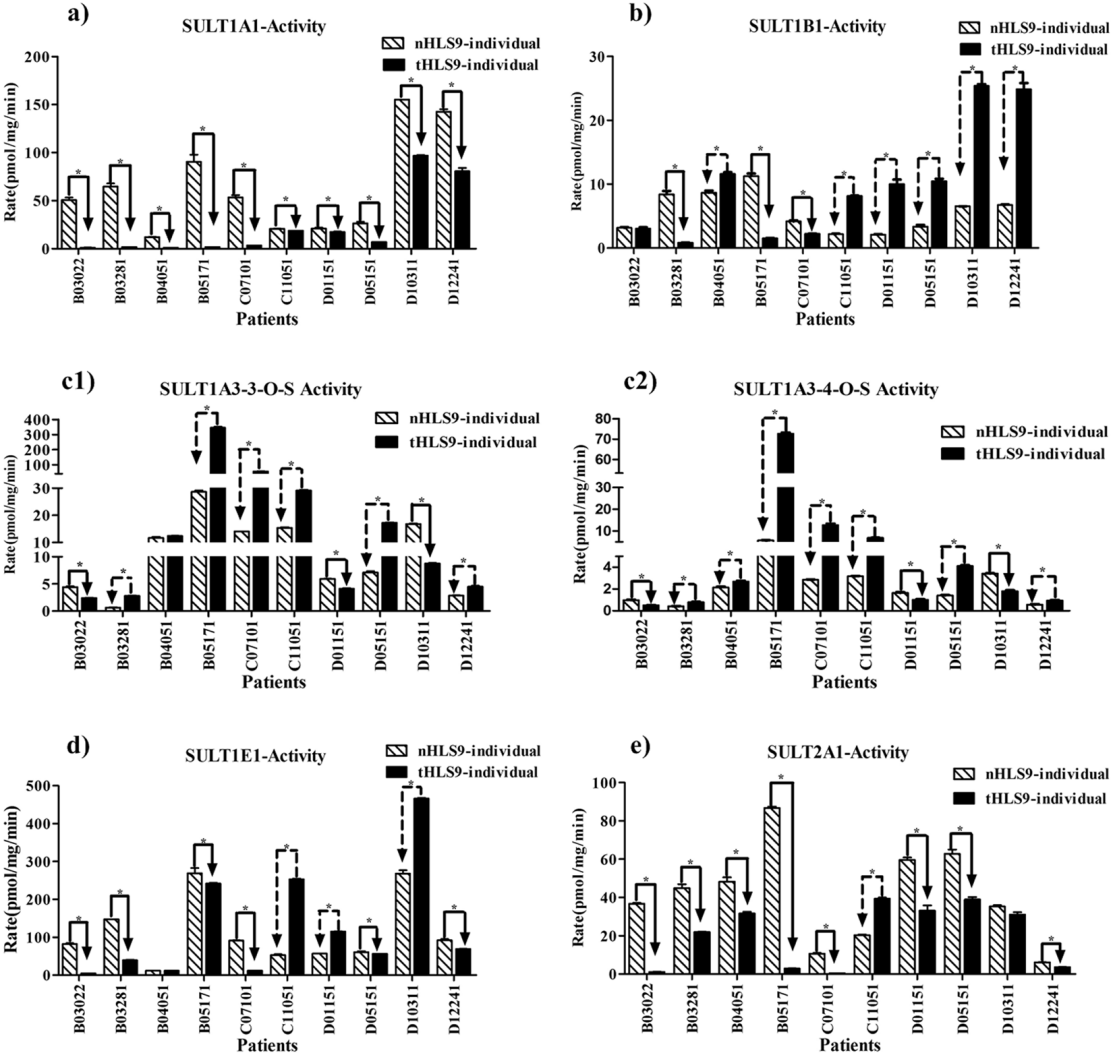
Activities of five SULT enzymes in human liver S9 prepared from HCC tumors (tHLS9-individual) and matched pericarcinomatous tissues (nHLS9-individual: (**a**–**e**). (**c1**) dopamine 3-O-sulfate, (**c2**) dopamine 4-O-sulfate. All the experiments were performed in triplicate, and the data are presented as mean ± SD. Paired sample *t-* test was performed for data analysis, and *P* < 0.05 was regarded as statistically significant.

We used the probe substrates at predetermined low concentrations (one concentration for each SULT: 1 μM p-nitrophenol for SULT1A1, 0.625 μM dopamine for SULT1A3, 2.5 μM 2-aminophenol for SULT1B1, 10 μM 17β-estradiol for SULT1E1 and 1 μM DHEA for SULT2A1) to measure SULT enzymatic activities in the hHLS9-individual, nHLS9-individual, and tHLS9-individual (Table 3). As shown in Fig. 4, for SULT1A1, the activities in pericarcinomatous S9 were all higher than those in tumor S9; for SULT1B1, 20% were higher in pericarcinomatous S9 than in tumor S9, 70% were lower in pericarcinomatous S9 than in tumor S9, and 10% were left unchanged in HCC patients; for SULT1A3, 30% were higher in pericarcinomatous S9 than in tumor S9, and 70% were lower in pericarcinomatous S9 than in tumor S9; for SULT1E1, 50% were higher in pericarcinomatous S9 than in tumor S9, 40% were lower in pericarcinomatous S9 than in tumor S9, and 10% were left unchanged; for SULT2A1, 80% were higher in pericarcinomatous S9 than in tumor S9, 10% were lower in pericarcinomatous S9 than in tumor S9, and 10% were left unchanged.

**Table 3:** SULTs Activities and Expression differences in Pericarcinomatous, Tumor and Healthy tissues.

Enzyme	No. of Quantified Donor	Probe Substrate Activities					Protein Amounts					mRNA Expression Levels				
		Mean	SD	Max	Min	Fold	Mean	SD	Max	Min	Fold	Mean	SD	Max	Min	Fold
Pericarcinomatous Tissues		pmol/mg/min					pmol/mg protein					relative gene expression rate				
^#^SULT1A1	10	63.7	50.9	155.2	12.0	12.9	13.2	10.3	33.6	2.8	12	0.9	0.2	1.0	0.6	1.6
^#^SULT1A3^a^	10	11.6	8.4	28.6	0.6	47.7	8.2	2.3	11.2	2.8	4	1.0	0.0	1.1	1.0	1.1
^#^SULT1A3^b^	10	2.2	1.6	5.7	0.4	14.2	8.2	2.3	11.2	2.8	4	1.0	0.0	1.1	1.0	1.1
^#^SULT1B1	10	5.7	3.1	11.3	2.1	5.4	7.6	2.7	11.8	2.6	4.5	1.0	0.1	1.2	0.8	1.5
^#^SULT1E1	10	113.2	89.0	269.1	12.1	22.2	1.2	0.4	1.7	0.6	2.8	1.1	0.1	1.2	1.0	1.2
^#^SULT2A1	10	41.1	24.9	86.7	6.2	13.9	42.4	24.0	77.0	12.6	6.1	1.0	0.0	1.1	1.0	1.1
Tumor Tissues																
SULT1A1	10	22.9	35.4	96.8	1.1	88	6.4	7.9	21.6	0.2	108	0.4	0.5	1.7	0.0	1.7
SULT1A3^a^	10	48.2	105.6	345.1	2.4	143.8	7.7	3.6	14.3	3.4	4.2	0.7	0.1	2.7	0.2	13.5
SULT1A3^b^	10	10.4	22.2	72.7	0.8	90.9	7.7	3.6	14.3	3.4	4.2	0.7	0.1	2.7	0.2	13.5
SULT1B1	10	9.8	8.9	25.4	0.8	31.8	12.2	4.9	20.9	6.4	3.3	1.0	0.4	1.6	0.6	2.7
SULT1E1	10	126.9	149.7	466.4	4.9	95.2	1.1	0.8	2.5	0.2	12.5	0.5	0.7	2.2	0.0	2.2
SULT2A1	10	20.4	16.6	39.4	0.4	98.5	29.9	24.2	73.1	3.4	21.5	0.2	0.3	1.1	0.0	1.1
Healthy Tissues																
SULT1A1	10	114.1	27.9	151.0	64.2	2.4	23.7	12.9	48.5	12.1	4.0	—	—	—	—	—
SULT1A3^a^	10	10.2	4.0	16.8	4.4	3.8	5.2	2.0	8.2	2.2	3.7	—	—	—	—	—
SULT1A3^b^	10	3.3	2.2	7.9	0.7	11.3	5.2	2.0	8.2	2.2	3.7	—	—	—	—	—
SULT1B1	10	4.2	1.7	7.1	2.2	3.2	5.4	2.2	8.8	1.8	4.9	—	—	—	—	—
SULT1E1	10	171.3	134.3	439.6	60.3	7.3	2.4	0.9	4.2	1.5	2.8	—	—	—	—	—
SULT2A1	10	76.80	7.7	89.7	66.0	1.4	63.6	19.9	90.9	31.1	2.9	—	—	—	—	—

^a^The metabolite is dopamine 3-O-Sulfate.

^b^The metabolite is dopamine 4-O-Sulfate.

^#^One concentration of probe substrate activities for each SULT: 1 μM p-nitrophenol for SULT1A1; 0.625 μM dopamine for SULT1A3; 2.5 μM 2-aminophenol for SULT1B1; 10 μM 17β-estradiol for SULT1E1 and 1 μM DHEA for SULT2A1.

### Impaired SULT enzymatic protein amounts in tumor tissues of HCC patients

**Figure 5:**
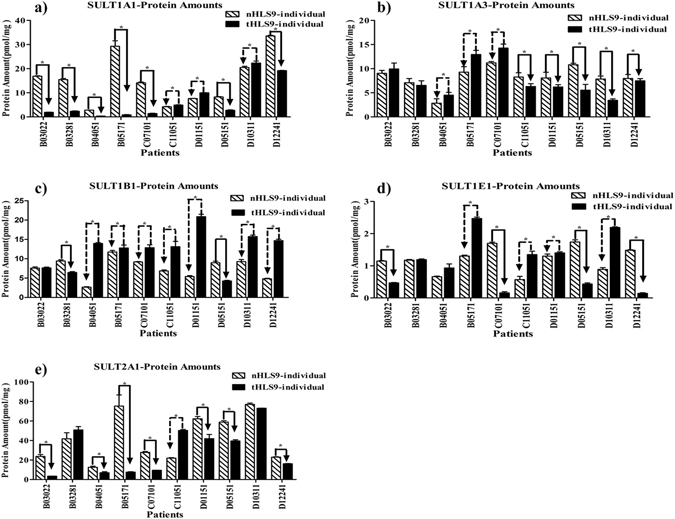
Protein amounts of the five SULT enzymes in human liver S9 prepared from HCC tumors (tHLS9-individual) and matched pericarcinomatous tissues (nHLS9-individual: (**a**–**e**). Absolute protein amounts were quantified through MRM LC-MS/MS method (n = 10). All the experiments were performed in triplicate, and data are presented as mean ± SD. Paired sample *t-*test was used for data analysis, and *P* < 0.05 was regarded as statistically significant.

For SULT1A1 isoform (as quantified by LC-MS/MS), 70% of the samples had higher amounts in pericarcinomatous tissues than tumor tissues, and 30% had lower amounts in pericarcinomatous tissues than tumor tissues; for SULT1B1, 20% had higher amounts in pericarcinomatous tissues than tumor tissues, 70% had lower amounts in pericarcinomatous tissues than tumor tissues, and 10% were left unchanged; for SULT1A3, 50% had higher amounts in pericarcinomatous tissues than tumor tissues, 30% had lower amounts in pericarcinomatous tissues than tumor tissues, and 20% were left unchanged; for SULT1E1, 40% had higher amounts in pericarcinomatous tissues than tumor tissues, 40% had lower amounts in pericarcinomatous tissues than tumor tissues, and 20% were left unchanged; for SULT2A1, 70% had higher amounts in pericarcinomatous tissues than tumor tissues, 10% had lower amounts in pericarcinomatous tissues than tumor tissues, and 20% were left unchanged (Fig. 5).

### Downregulated SULT enzymatic mRNA expression levels in tumor tissues of HCC patients

**Figure 6:**
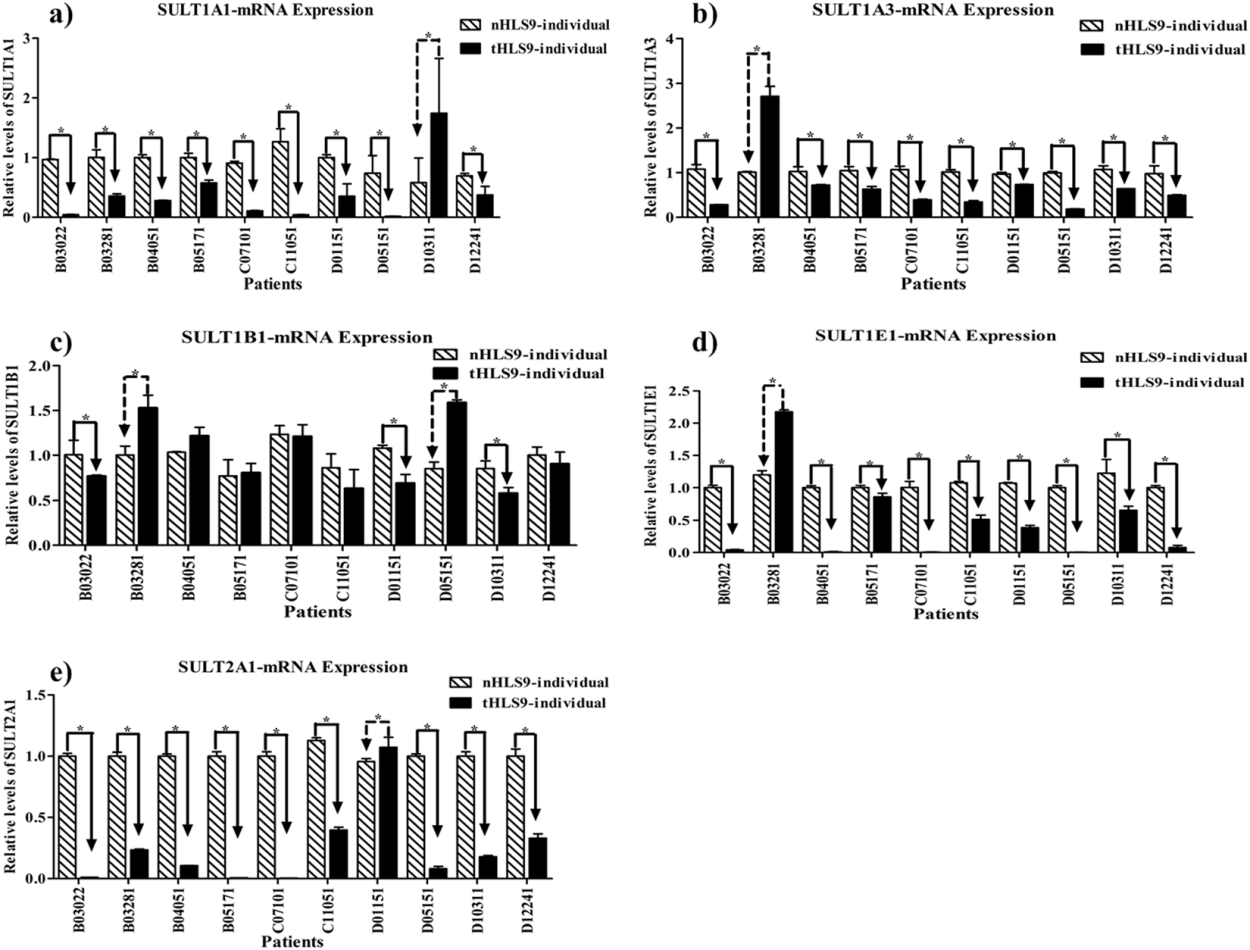
mRNA expressions of the five SULT enzymes in tumors and matched pericarcinomatous tissues of 10 human liver S9 samples with HCC (**a**–**e**). Relative expression differences were calculated through the comparative ΔΔCt method, and Ct values were normalized to GAPDH expression levels. All measurements were performed in triplicate and the relative expression levels of the target genes were presented as 2^−ΔΔCt^. Data are presented as mean ± SD. Paired-sample *t-*test was used for data analysis, and *P* < 0.05 was regard as statistically significant.

The mRNA expression levels of SULT enzymes were determined in tumors and matched pericarcinomatous tissues of 10 HCC patients using real-time PCR. As shown in Fig. 6, the SULT1A1, SULT1A3, SULT1B1, SULT1E1 and SULT2A1 were unequivocally expressed in all the samples of the HCC tumors and matched pericarcinomatous tissues. The mRNA expression levels of SULT1A1 (Fig. 6(a)), SULT1A3 (Fig. 6(b)), SULT1E1 (Fig. 6(d)), and SULT2A1 (Fig. 6(e)) decreased or drastically decreased in 90% of the tumor samples in comparison with the matched pericarcinomatous samples. SULT1B1 expression showed significantly small differences, with a few matched samples showing any significant difference in RNA expression (Fig. 6(c)).

### Correlation of protein amounts and mRNA expression levels with SULT enzymatic activities

To determine whether protein amounts are more effective than mRNA levels as indicators for predicting SULT activities in liver tissues, we correlated the protein amounts and mRNA expression levels with the activities of the SULT enzymes. As shown in Table 4, in tumor tissues, correlation coefficients greater than 0.593 were found between protein amounts and enzymatic activities in all the measured SULTs, particularly SULT1A1, which showed a high correlation coefficient of 0.950. In pericarcinomatous tissues, the correlation coefficient values of more than 0.6 were found in three SULTs (1A1, 1B1, and SULT1E1), and SULT1A1 showed a high correlation coefficient of 0.849. No correlation was found between protein amounts and activities of the SULT1A3 and SULT1B1 in pericarcinomatous tissues. For all the measured SULTs in the pericarcinomatous and tumor tissues, correlations were not found between protein amounts and mRNA expression levels (except for SULT1A1 and SULT1B1 in tumor tissues). In summary, in the pericarcinomatous and tumor tissues, the correlation between the SULT enzymatic activities and the protein amounts was better than that between the activities and the mRNA expression levels for a majority of the SULT enzymes.

**Table 4:** Correlation of Enzyme Activity Protein to Protein Amounts and mRNA Expression Level of SULT Enzymes.

Enzyme	Protein Amounts VS Activity		Protein Amounts VS mRNA		Activity VS mRNA	
	N^c^	T^d^	N^c^	T^d^	N^c^	T^d^
	r^2^	r^2^	r^2^	r^2^	r^2^	r^2^
SULT1A1	0.849^e^	0.977^e^	0.486	0.705^e^	0.698^e^	0.730^e^
SULT1A3^a^	0.120	0.593	0.159	0.166	0.422	0.402
SULT1A3^b^	0.148	0.599	0.159	0.159	0.092	0.095
SULT1B1	0.222	0.412	0.386	0.676^e^	0.292	0.328
SULT1E1	0.690^e^	0.783^e^	0.362	0.479	0.474	0.197
SULT2A1	0.637^e^	0.690^e^	0.366	0.381	0.351	0.419

^a^The metabolite is dopamine 3-O-Sulfate.

^b^The metabolite is dopamine 4-O-Sulfate.

^c^N: Pericarcinomatous.

^d^T: Tumor.

^e^Statistical significance of association (P < 0.05).

^r2^Pearson correlation coefficient.

### Non-impairment of catalytic capacity of SULT enzymes was in healthy, pericarcinomatous, and HCC tumor S9

**Figure 7:**
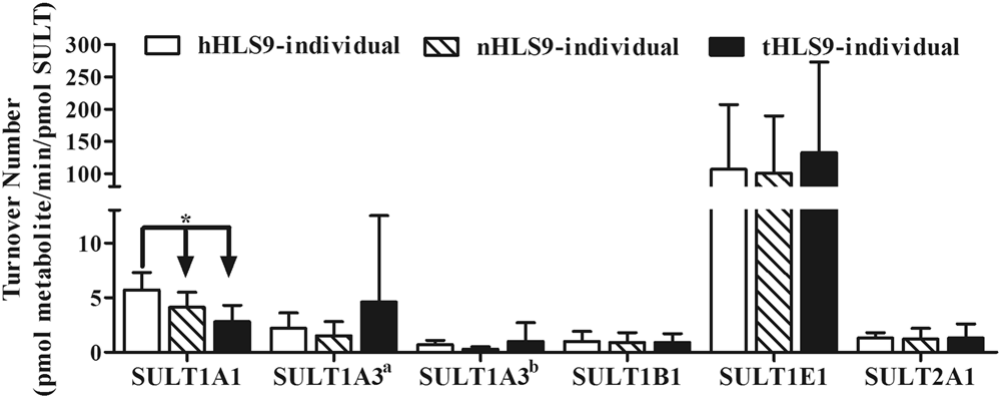
TONs of the probe substrates metabolized by SULT enzymes in healthy donors, HCC tumors, and match pericarcinomatous tissues, thereby, indicating the catalytic efficiency of enzymes. There is one concentration of probe substrate activities for each SULT, as follow: 1 μM p-nitrophenol for SULT1A1, 0.625 μM dopamine for SULT1A3, 2.5 μM 2-aminophenol for SULT1B1, 10 μM 17β-estradiol for SULT1E1, and 1 μM DHEA for SULT2A1. It is defined as the number of substrate molecules that can be converted by enzyme proteins into products per unit of time. Paired sample *t*-test or Wilcoxon’s sign rank test was used for data analysis. “*”Denotes statistical significance (*P* < 0.05).

As shown in Fig. 7 and Table 5, the TONs of SULTs other than SULT1A1 in the tumor tissues were nearly identical to the values observed in the pericarcinomatous tissues and healthy samples (*P* > 0.05), thereby suggesting that the catalytic efficiency of SULTs was not seriously impaired in tumor S9. For SULT1A1, the catalytic efficiency of SULT1A1 was significantly higher in the healthy human samples than in the pericarcinomatous and tumor tissues, but the maximal difference was considerably moderate at only 1.9-fold (healthy tissues vs. tumor tissues) or less (healthy tissues vs. pericarcinomatous tissues).

**Table 5:** Summary of turnover numbers of probe substrate metabolized by SULT enzymes.

Isoforms	TON (hHLS9-individual)^a^			TON (nHLS9-individual)^b^			TON (tHLS9-individual)^c^			n^d^
	Mean	SD	95%CI^e^	Mean	SD	95%CI	Mean	SD	95%CI	
**pmol metabolite formed/min/pmol SULT enzymes**										
SULT1A1	5.4	1.5	4.4~6.5	4.1^f^	1.4	3.1~5.1	2.8^f^	1.5	1.8~4.0	10
SULT1A3^g1^	2.2	1.4	1.2~3.3	1.5	1.3	0.5~2.4	4.6	7.9	1.1~10.2	10
SULT1A3^g2^	0.7	0.4	0.4~0.9	0.3	0.2	0.1~0.5	1.0	1.7	0.2~2.2	10
SULT1B1	1.0	0.9	0.4~1.6	0.9	0.9	0.3~1.6	0.9	0.8	0.3~1.4	10
SULT1E1	106.6	100.3	34.9~178.4	100.9	88.9	37.3~164.5	132.4	140.31	32.0~232.8	10
SULT2A1	1.3	0.5	0.97~1.7	1.2	1.0	0.4~1.9	1.3	1.3	0.4~2.2	10

^a,b,c^Turnover numbers of probe substrates metabolized by SULT enzymes in hHLS9-individual, nHLS9-individual and tHLS9-individual, which were calculated as the ration of maimal metabolic rate (Vmax) to the total amount of SULTs enzyme that is shown in Figs 4 and 6.

^d^Numbers of donors were calculating turnover numbers.

^e^95% CI represents the 95% confidence interval. Paired-samples *t* test or Wilcoxon sign rank test was used for data analysis.

^f^Statistical significance (*P* < 0.05).

^g1,g2^Dopamine was the probe substrate of the SULT1A3. Dopamine has two isomeride metabolites: dopamine 3-O-Sulfate and dopamine 4-O-Sulfate.

## Discussion

The results of our study clearly showed that the rapid LC-MS/MS method was superior in speed for all SULT isoforms and in specificity for a majority of the SULT isoforms in determining the levels of SULT protein amounts and activities. This superiority is supported by the following: the LC-MS/MS method was more specific than Western blot in determining the protein amounts of SULTs, the protein amounts obtained from the LC-MS/MS method were representative of the probe substrate activities in the human liver S9 samples of pericarcinomatous and HCC patients, and the method was run in less than 10 min with a maximum sample preparation time of 6 h and may thus be readily used in human clinical situations. The latter may aid the development of a laboratory SULT quantification method useful for precision medicine with respect to drugs that mainly undergo sulfonation in both healthy and HCC populations. The actual implementation would probably require a prospective clinical study.

Our results also showed that the LC-MS/MS method for detecting SULT enzymes with respect to specificity, accuracy and speed is superior to the current methods for detecting the expression levels of SULT enzymes based on Western blot or RT-PCR. For SULT enzymes, the mRNA expression levels cannot reflect protein expression, and Western blot analysis involves long and complex experimental procedures that may be compromised because of the lack of highly specific antibodies^33^. By contrast, the precision and accuracy of the new method meet strict quantification standards, for the determination of CYP and UGT protein amounts in human liver microsomes in a previous study^22^ and in other research laboratories^34, 35^. The robust method is reproducible and reliable.

Large amounts of data demonstrated that protein (SULT) amounts as measured using LC-MS/MS were more representative of the probe substrate activities than the expression levels derived from q-PCR or some Western blot. The advantages of the LC-MS/MS method include its specificity, whereas antibodies are only specific 40% of the time. For SULT1A1, the activities and expression levels were the highest, and for some unknown reason, the lack of specificity did not seem to negatively affect the correlation as much. In our previous study, we also observed that for a few abundantly expressed CYP isoforms (e.g., CYP3A4), the correlation coefficients between various measurements were highly satisfactory^22^.

The LC-MS/MS method may be applied directly in clinical detection, because only small amounts of tissues are required for simultaneously quantifying the protein amounts of five major SULT enzymes in human liver S9. In fact, we and other researchers could assay both phase I and phase II enzymes in a single run, reaching 50 enzymes and transporters combined^19, 22, 35^. Thus, the MRM UHPLC-MS/MS method is capable of distinguishing multiple factors that govern drug disposition in a single run, thereby allowing the data generated to be utilized for modifying drug intervention strategies in clinical settings. For patients suffering from inoperable HCC, a liver biopsy followed by LC-MS/MS-based SULT enzyme quantification may be a good starting point for the development of personalized chemotherapy or chemoablation therapy. Ideally, selecting drugs on the basis of HCC SULT levels could identify drugs that are highly active in tumors but less toxic or nontoxic in normal liver cells.

When a superior analytical method is developed, it could potentially lead to an improved understanding of the physiology or pathophysiology of the organ system. In this case, our systemic studies showed that drug metabolism via SULTs is often altered, but the extent of the changes is highly individualized, with some tumor S9 exhibiting a significant decrease in protein amounts and probe substrate activities and with others exhibiting a significant increase relative to matched pericarcinomatous tissues. Theoretically, such a large change may be due to changes in expression levels or catalytic efficiency of each expressed enzyme (due to post-translational modification). Using TONs of SULTs as a measurement of catalytic efficiency, we found that with the exception of that for SULT1A1, TON did not change in the tumor vs. pericarcinomatous tissues, even with a large decrease in activities or expressions in 90% of the subjects. For SULT1A1, the change of its TON was relatively small (less than twofold). Hence, the data showed that the tumor cells usually expressed lower amounts of SULT proteins in comparison with pericarcinomatous tissues, but those SULT proteins appeared to be just as active, with the exception of SULT1A1, which appeared to be moderately less active. Further studies are needed to determine why SULT1A1 had a low TON in tumor tissues.

Lastly, SULTs are involved in the metabolism of carcinogens and drugs^36, 37^. A recent report further suggests SULT1A3 may be used as prognostic biomarkers of osteosarcoma^38^. Whether finding applies to liver cancer is unknown. What we did show is that in terms of activity levels, the probe substrate activities of SULT1A3 were significantly higher in most of the tumor samples and in pooled tumor S9 preparation. Hence, SULT1A3 overexpression may also be an indicator of liver cancer prognosis, although we did not find direct evidence linking them together. In addition, the probe substrates for SULTs do not always enjoy the same status as CYP probe substrates; that is, they might not be as carefully studied as fewer labs are studying SULT activities in comparison with those exploring CYPs. Hence, caution must be exercised when linking probe substrate activities with expressions of SULT isoform exclusivity.

## Conclusion

This study is the first to demonstrate that the absolute quantification of SULT enzymes through MRM UHPLC-MS/MS can be achieved using human liver S9 samples. We believe that our method features several advantages, including higher specificity, better reproducibility, and better precision in comparison with Western blot and q-PCR. Moreover, the short run time means the method can rapidly analyze many SULT isoforms in one MRM assay in comparison with traditional methods. In this way, this can be used in the simultaneous quantification of multiple enzymes and efflux transporters from a single patient sample in a relatively short period of time.

## Electronic supplementary material

LC-MS/MS quantification of sulfotransferases is better than conventional immunogenic methods in determining human liver SULT activities: implication in precision medicine
