# Supplementary material for: LC-MS/MS quantification of sulfotransferases is better than conventional immunogenic methods in determining human liver SULT activities: implication in precision medicine
Source: Sci Rep. 2017 Jun 20;7:3858. doi: 10.1038/s41598-017-04202-w (PMC5478605; doi:10.1038/s41598-017-04202-w)
Supplement: Supplementary file 1 — LC-MS/MS quantification of sulfotransferases is better than conventional immunogenic methods in determining human liver SULT activities: implication in precision medicine [file 41598_2017_4202_MOESM1_ESM.pdf]

Supplementary Information For

LC-MS/MS quantification of sulfotransferases is better than conventional immunogenic methods in determining human liver SULT activities: implication in precision medicine

Cong Xie[1, 2#], Tong-meng Yan[2, 5#], Jia-mei Chen[2], Xiao-yan Li[2] , Juan Zou[2], Li-jun Zhu[2], Linlin Lu[2] , Ying Wang[2] , Fu-yuan Zhou[3, 2]*, Zhong-qiu Liu[1, 2]*, Ming Hu[4]*

[1]Department of Pharmaceutics, School of Pharmaceutical Sciences, Southern Medical University, Guangzhou, Guangdong, China, 510515.

[2]International Institute for Translational Chinese Medicine, Guangzhou University of Chinese Medicine, Guangzhou, Guangdong, China, 510006.

[3]Department of Infectious Diseases, Nanfang Hospital, Southern Medical University, Guangzhou, Guangdong, China, 510515.

[4]Department of Pharmacological and Pharmaceutical Sciences, College of Pharmacy, University of Houston, Houston, TX 77030, USA.

[5]State Key Laboratory of Quality Research in Chinese Medicine, Macau University of Science and Technology, Macau (SAR), China.

Table of Contents:

Table S1. Characteristics of peptides for quantification of SULTs

Table S2. MRM transitions of peptides for quantifying SULTs enzymes

Table S3. Incubation Conditions for SULT Activity Assays

Table S4. Analytical Parameters for Activity Assays of SULTs

Table S5. PCR Primers for Human SULT Enzymes and GADPH

Table S6. Protein expression levels of SULT enzymes in human liver S9 fraction

Table S7. Intra-and inter-day precision and accuracy for the determination of SULTs

Table S8. Extraction recovery of signature peptides for quantifying SULTs enzymes

Table S9. Validation for LC-MS/MS quantification of protein amount of SULTs using standard addition method

Figure of Contents:

Fig. S1.The procedure of selecting the unique peptides for the MRM UHPLC-MS/MS.

Fig. S2. The peptide of the MS/MS spectra of SULT1A1, SULT1A3, SULT1B1, SULT1E1 and SULT2A1, in respectively, obtained from ESI-MS in positive. ionization mode.

Fig. S3. The MS^2 spectrograms of prototypic peptides measured in the present study.

Fig. S4. Standard curves of prototypic peptides for quantifying SULT enzymes.

Fig. S5. Western blot analysis the protein expression levels of 5 SULTs enzymes in healthy human liver S9 from 10 individual donors.

Table S1. Characteristics of peptides for quantification of SULTs enzymes.

Isoforms	Sequence	Length （AA）	Mass（Da）	Unipro Entry
SULT1B1	$V_{168}AYGSWFTHVK_{178}$	11	1293.65	O43704
SULT1E1	$L_{39}IHFLER_{45}$	7	926.53	Q53X91
SULT2A1	$W_{34}IQSVPIWER_{44}$	10	1304.69	A8K015
SULT1A2	$V_{148}YPHPGTWESFLEK_{161}$	14	1688.82	E9PKR8
SULT1A1	$T_{107}HLPLALLPQTLLDQK_{122}$	16	1800.05	H3BRY5
SULT1A3	$A_{74}HPEPGTWDSFLEK_{87}$	14	1612.75	H3BPL6

Table S2. MRM transitions of peptides for quantifying SULT enzymes.

Isoforms	Quantitative Ions			Qualitative Ions		
	MRM Transitions	Ion Series[a]	CE[b]	MRM Transitions	Ion Series[a]	CE[b]
SULT2A1	657.5>700.5	y5	16	657.5>1014.3	y8	20
	657.5>272.3	a2	20	657.5>886.5	y7	20
SULT1A1	601.1>471.6	y5[*]	8	601.1>859.8	b8	12
	601.1>746.3	b7	16			
SULT1A2	564.1>714.4	y12	8	564.1>263.0	b2	8
	564.1>225.2	a2	20	564.1>666.2	a11	16
SUT1A3	538.8>532.6	b5	4	538.8>290.0	b2	20
	538.8>690.0	b7	8	538.8>738.8	y6	8
SULT1B1	432.2>562.8	y6	8	432.2>158.7	b3	24
	432.2>171	b2	12			
SULT1E1	309.8>408.0	y6	12	309.8>175.1	y1	12
	309.8>564.2	y4	12			
Pep-IS	565.0>398.2	y^{03}	12	565.0>221.1	b2	26
	565.0>795.4	y7	15	565.0>454.9	y^{8+}	22

[a]: The nomenclature, reported by Roepstorff [1] and Johnson [2] for fragment ions in mass spectrum of signature peptides was used.

[b]: CE: collision energy

Table S3. Incubation conditions for SULT activity assays.

Isoform	Probe Substrate	Substrate Conc.[a] (μM)	Substrate Range [b] (μM)	Protein Conc. (mg/ml)	Incubation Time (min)	Metabolite
SULT1A1	p-Nitrophenol	1	0.125-40	0.25	30	Potassium 4-nitrophenyl sulfate
SULT1A3[#]	Dopamine	0.625	0.25-300	0.5	30	Dopamine 3-O-Sulfate
SULT1A3[#]	Dopamine	0.625	0.25-300	0.5	30	Dopamine 4-O-Sulfate
SULT1B1	2-Aminophenol	2.5	0.25-40	0.25	30	2-aminophenol sulfate
SULT1E1	17β-estradiol	10	0.3125-40	0.25	30	17β-estradiol 3-sulfate
SULT2A1	DHEA[c]	1	0.125-20	0.25	30	Dehydroisoandrosterone 3-sulfate sodium

a: A specific concentration of substrate was used for determining SULT activity of each patient.

b: A range concentration of substrate was used for determining kinetic profile of SULT in human liver S9.

c: DHEA: Dehydroisoandrosterone

[#]: Dopamine was the probe substrate of the SULT1A3. Dopamine has two isomeride metabolites in phase II

Table S4. Analytical parameters for activity assays of SULTs.

Parameters (LC-MS/MS)	SULT1A1	SULT1A3	SULT1B1	SULT1E1	SULT2A1
Analyte	p-Nitrophenol	Dopamine	2-Aminophenol	17β-estradiol	DHEA[b]
Injection volume(μl)	1	1	1	1	1
Mobile phase(A/B)	Ammonium acetate /ACN[a]	Ammonium acetate /ACN	Ammonium acetate /ACN	Ammonium acetate /ACN	Ammonium acetate /ACN
Gradient program (%B(min))	20(0) →45(2) →45(6) →20(6.1-7)	5(0-2) →70(3.9) →70(7) →5(7.1-8)	5(0) →45(2) →45(5) → 5(5.1-6)	20(0) →45(2)→ 45(4) →20(4.1-5)	20(0) →45(2)→ 45(4) →20(4.1-5)
Analyte transition	217.97>138	231.8>151.90	188>108	351.10>271.20	367.10>97
Collision energy(V)	8	20	20	32	48
Cell accelerator voltage(V)	4	5	2	5	4
Retention time (min)	3.851	2.330[#] 2.720[#]	1.543	2.443	2.637
Internal standard	PS[c]	PS	PS	PS	PS

a:ACN:acetonitrile. #: The retention time 2.330 is the dopamine-4-O-Sulfate and the 2.720 is the dopamine-3-O-Sulfate.

b: DHEA: Dehydroepiandrosterone c: pregnenolone sulfate

Table S5. PCR primers for human SULT enzymes and GADPH.

GAPDH	forward	GGCCTCCAAGGAGTAAGACC
	reverse	AGGGGAGATTCAGTGTGGTG
SULT1A1	forward	AAAGCCCCAGGGATTCCCTCA
	reverse	GGAAACTGCCACATCCTTTGCGT
SULT1A3	forward	CGATGCGGACTATGCGGAGAAG
	reverse	GACATGAGCCACTGTGCCTGAC
SULT1B1	forward	GCTGGTGACTGGAAGAATTACT
	reverse	GAAGAGCCTGTGGTTACATTGT
SULT1E1	forward	GGCTGGTCATCCAAATCCTGG
	reverse	AGGAACCATAAGGAACCTGTCC
SULT2A1	forward	TCGTGATAAGGGATGAAGATGTAATAA
	reverse	TGCATCAGGCAGAGAATCTCA

Table S6 Protein expression levels of SULT enzymes in human liver S9 fraction.

The expression of enzymes was determined in human liver S9 from 10 donors. Each value represents the mean ± SD (n=10).

Enzyme	No. of Quantified Donor	Protein Amounts				Relative Protein Expression Level				Correlation ($^{a}r^2$)
		Mean	Max	Min	Max/Min	Mean	Max	Min	Max/Min	
		pmol/mg protein				relative protein expression level/ GAPDH				
SULT1A1	10	23.65±12.93	48.54	12.14	4.0	0.55±0.31	1.06	0.24	4.4	0.899[b]
SULT1A3	10	5.18±2.04	8.79	2.2	4.0	0.33±0.07	0.44	0.24	1.8	0.492
SULT1B1	10	5.39±2.22	8.82	1.81	4.9	0.21±0.05	0.29	0.14	2.1	0.764[b]
SULT1E1	10	2.39±0.94	4.19	1.51	2.8	0.49±0.16	0.65	0.20	3.2	0.691[b]
SULT2A1	10	63.56±19.89	90.99	31.12	3.0	0.46±0.13	0.69	0.28	2.5	0.546

Max: maximum, Min: minimum, we used the isotope label-free LC-MS/MS to quantify protein amounts of the SULTs. And we used the western blot analysis to semi-quantify the relative protein expression levels of the same SULTs in the human liver S9 prepared from the 10 donor.

a r^2: Pearson or Spearman correlation coefficient

b: Statistical significance of association (*P*<0.05)

Table S7. Table 4 Intra-and inter-day precision and accuracy for the determination of SULTs

Isoforms	LLOQ（nM）	Accuracy（%Deviation）			Intra-day Precision(%RSD)			Inter-day Precision(%RSD)		
		Low	Mid	High	Low	Mid	High	Low	Mid	High
SULT2A1	0.3	3.0	5.7	6.7	-4.5	-9.6	-1.3	-3.6	-13.9	-3.8
SULT1A1	0.4	6.0	8.3	2.6	-0.7	-7.4	1.1	-1.2	-8.9	-1.2
SULT1A3	0.5	4.0	5.4	5.4	-10.8	8.5	-2.5	-9.2	9.6	-7.0
SULT1B1	0.8	12.5	7.5	5.2	-10.3	-16.2	-11.0	-18.1	-16.7	-9.4
SULT1E1	0.3	4.4	3.5	2.6	-11.5	-10.6	0.5	-16.1	-11.0	-1.2

Precision and accuracy were which for the determination of SULT isoforms. Intra- and Inter-Day precision and accuracy were determined by measuring standard samples at three concentration levels (Low, Mid, High): 1.56, 12.5, 100nM for all the signature peptides.

Table S8: Extraction recovery of signature peptides for quantifying SULTs enzymes

Isoforms	Recovery（%）（mean±SD，n=3）		
	Low	Mid	High
SULT2A1	106.2±20.3	84.3±5.1	87.8±4.3
SULT1A1	109.7±6.1	92.5±3.9	107.3±1.5
SULT1A3	97.9±6.3	79.7±6.6	80.9±8.5
SULT1B1	80.2±10.5	81.2±1.8	88.7±4.2
SULT1E1	85.9±4.8	93.1±5.0	98.6±1.8

Table S9: Validation for LC-MS/MS quantification of protein amount of SULTs using standard addition method

Isoforms	Amount in HLS9 (mean ±SD,n=4,pmol/sample)	Spiked amount of peptide (pmol/mg)	Calculated amount of spiked peptide (pmol/mg)	Inaccuray (%Deviation)
SULT2A1	23.6 ±2.5	29.6	6.0	4%
SULT1A1	17.8 ±1.0	24.1	6.3	2%
SULT1A3	5.9 ±0.7	11.4	5.5	12%
SULT1B1	5.2 ±0.2	10.7	5.6	11%
SULT1E1	0.9 ±0.0	7.1	6.2	1%

Supplementary Figures

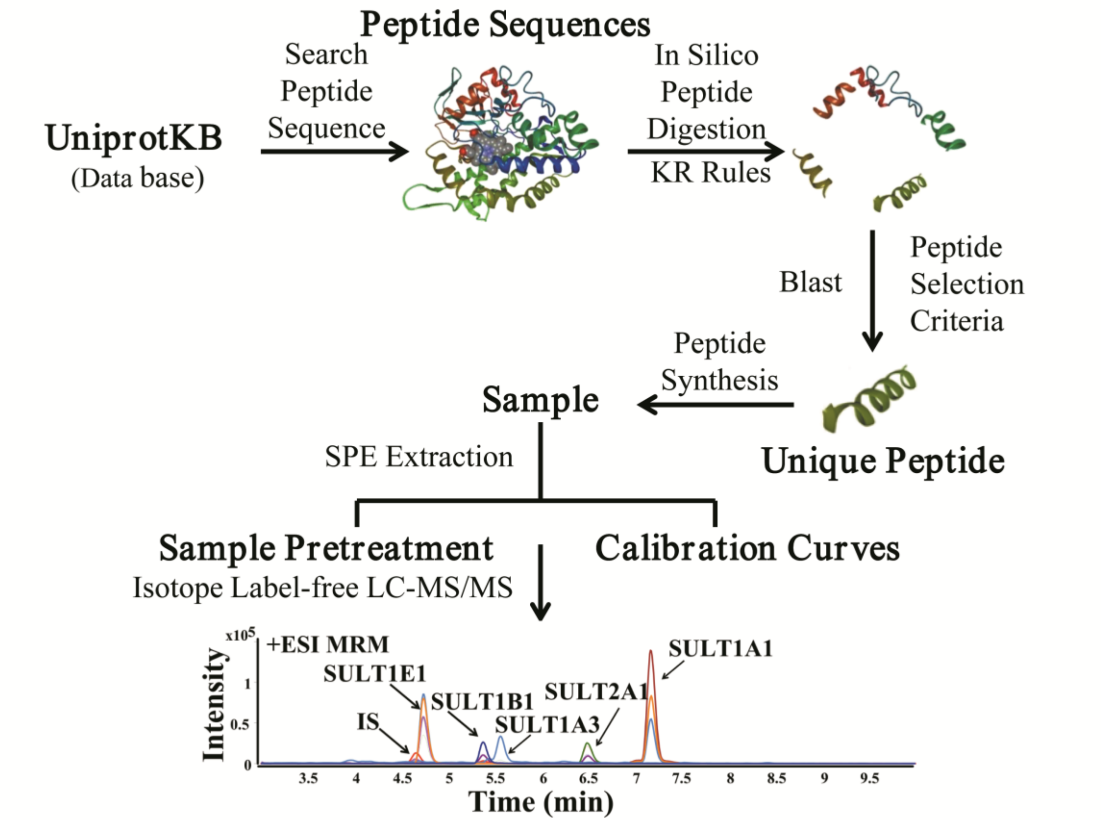

Figure S1. The full protein sequences for the SULT1A1, SULT1A2, SULT1A3, SULT1B1, SULT1E1 and SULT2A1 were obtained in batches from UniprotKB database and take them in the silico peptide digestion and excluded theirs' regions of uncertainty. The uniqueness of peptide sequences was verified by using BLAST search analysis. And, we developed the isotope label-free UHPLC-MS/MS for the quantification of SULTs in human liver S9.

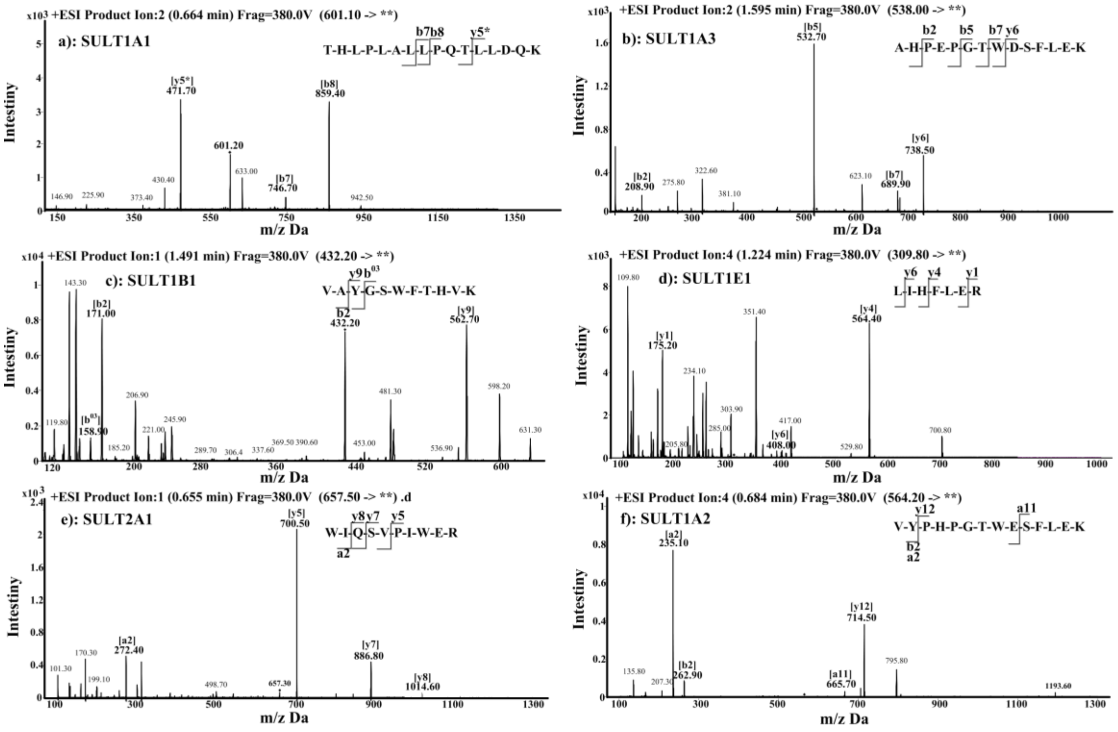

Figure S2. The peptide of the MS/MS spectra of m/z 601.1, +3-charged THLPLALLPQTLLDQK, m/z 538.8, +3-charged AHPEPGTWDSFLEK, m/z 432.2, +3-charged VAYGSWFTHVK, m/z 309.8, +3-charged LIHFLER, m/z 657.5, +2-charged WIQSVPIWER, m/z 564.1, +3-charged VYPHPGTWESFLEK, which were representative of the (a) SULT1A1, (b) SULT1A3, (c) SULT1B1, (d) SULT1E1, (e) SULT2A1, (f) SULT1A2 in respectively, obtained from ESI-MS in positive ionization mode. Arrows indicate optimum product ions for use in MRM transitions.

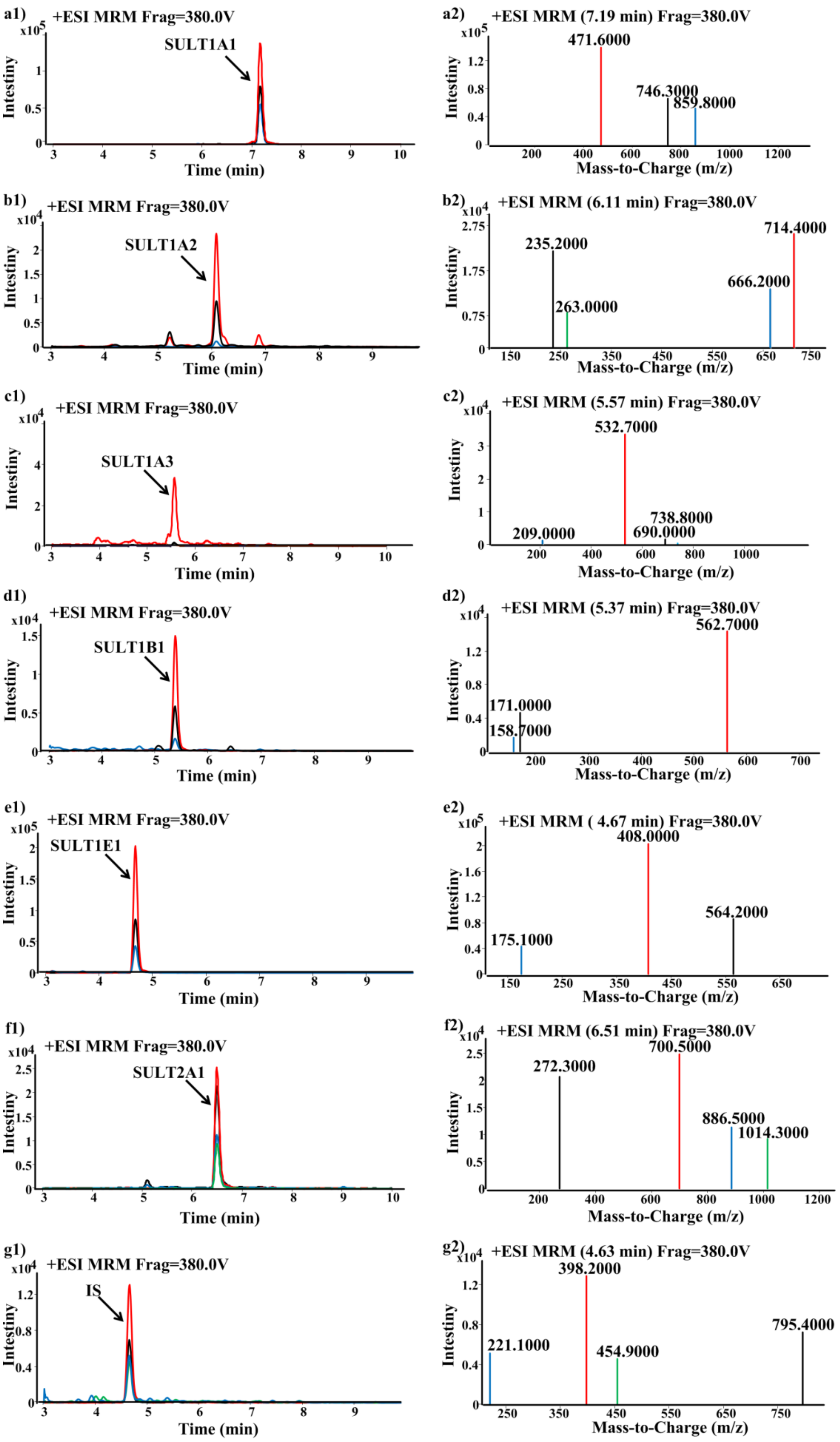
a1)
+ESI MRM Frag=380.0V
x10^5
SULT1A1
Intestiny
Time (min)
a2)
+ESI MRM (7.19 min) Frag=380.0V
x10^5
471.6000
746.3000
859.8000
Intestiny
Mass-to-Charge (m/z)
b1)
+ESI MRM Frag=380.0V
x10^4
SULT1A2
Intestiny
Time (min)
b2)
+ESI MRM (6.11 min) Frag=380.0V
x10^4
235.2000
263.0000
666.2000
714.4000
Intestiny
Mass-to-Charge (m/z)
c1)
+ESI MRM Frag=380.0V
x10^4
SULT1A3
Intestiny
Time (min)
c2)
+ESI MRM (5.57 min) Frag=380.0V
x10^4
532.7000
209.0000
738.8000
690.0000
Intestiny
Mass-to-Charge (m/z)
d1)
+ESI MRM Frag=380.0V
x10^4
SULT1B1
Intestiny
Time (min)
d2)
+ESI MRM (5.37 min) Frag=380.0V
x10^4
562.7000
171.0000
158.7000
Intestiny
Mass-to-Charge (m/z)
e1)
+ESI MRM Frag=380.0V
x10^5
SULT1E1
Intestiny
Time (min)
e2)
+ESI MRM (4.67 min) Frag=380.0V
x10^5
408.0000
564.2000
175.1000
Intestiny
Mass-to-Charge (m/z)
f1)
+ESI MRM Frag=380.0V
x10^4
SULT2A1
Intestiny
Time (min)
f2)
+ESI MRM (6.51 min) Frag=380.0V
x10^4
700.5000
272.3000
886.5000
1014.3000
Intestiny
Mass-to-Charge (m/z)
g1)
+ESI MRM Frag=380.0V
x10^4
IS
Intestiny
Time (min)
g2)
+ESI MRM (4.63 min) Frag=380.0V
x10^4
398.2000
795.4000
221.1000
454.9000
Intestiny
Mass-to-Charge (m/z)

Figure S3. The a1-g1 showed that MRM chromatograms of SULT1A1, SULT1A2, SULT1A3, SULT1B1, SULT1E1, SULT2A1 and Internal Standard (IS). The a2-g2 showed that MS^2 spectrograms of prototypic peptides. The red bar graph was the highest sensitivity transition for the quantitative ion.

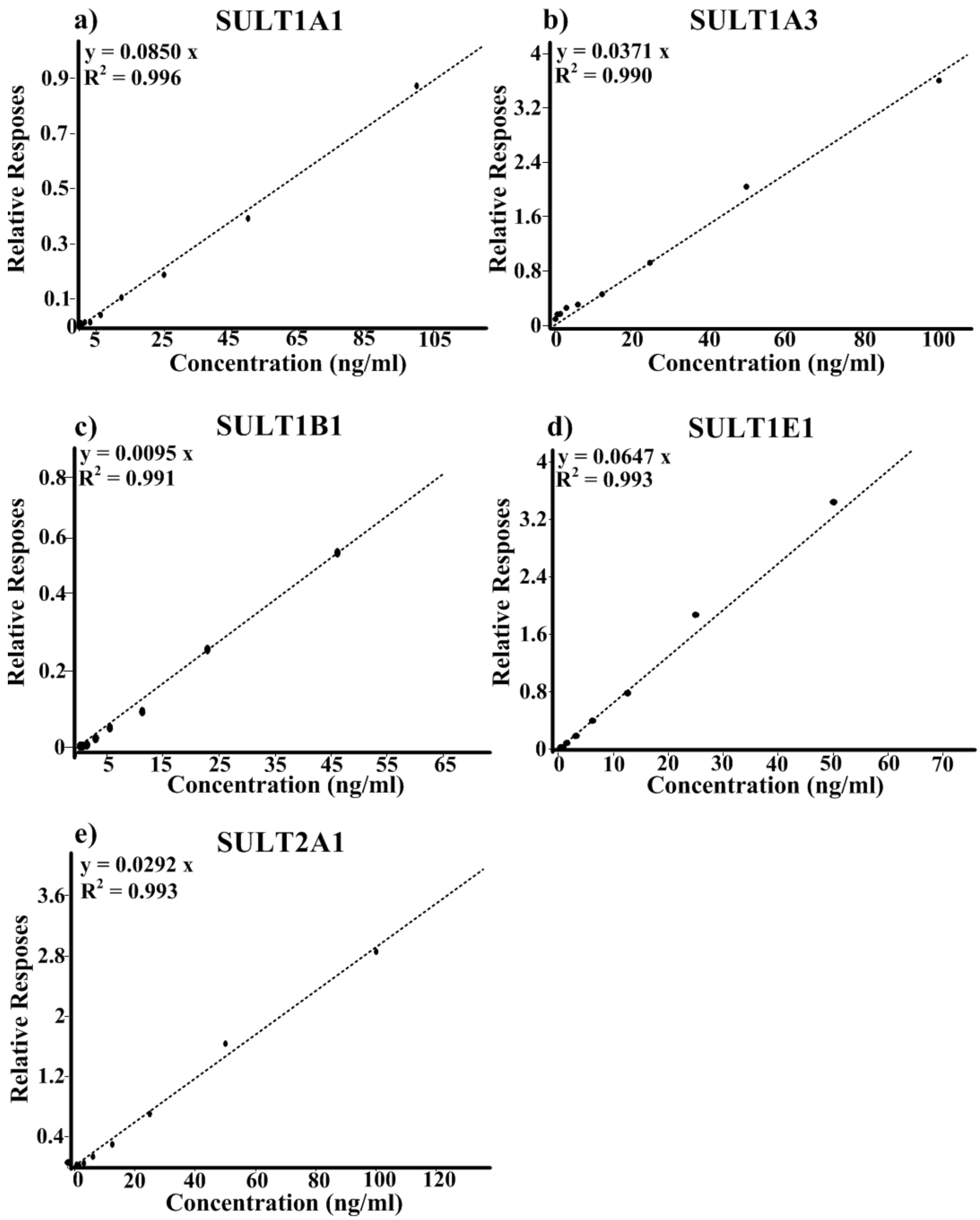

Figure. S4. Standard curves of prototypic peptides for quantifying SULT enzymes, which were representative of the (a) SULT1A1, (b) SULT1A3, (c) SULT1B1, (d) SULT1E1 and (e) SULT2A1, respectively.

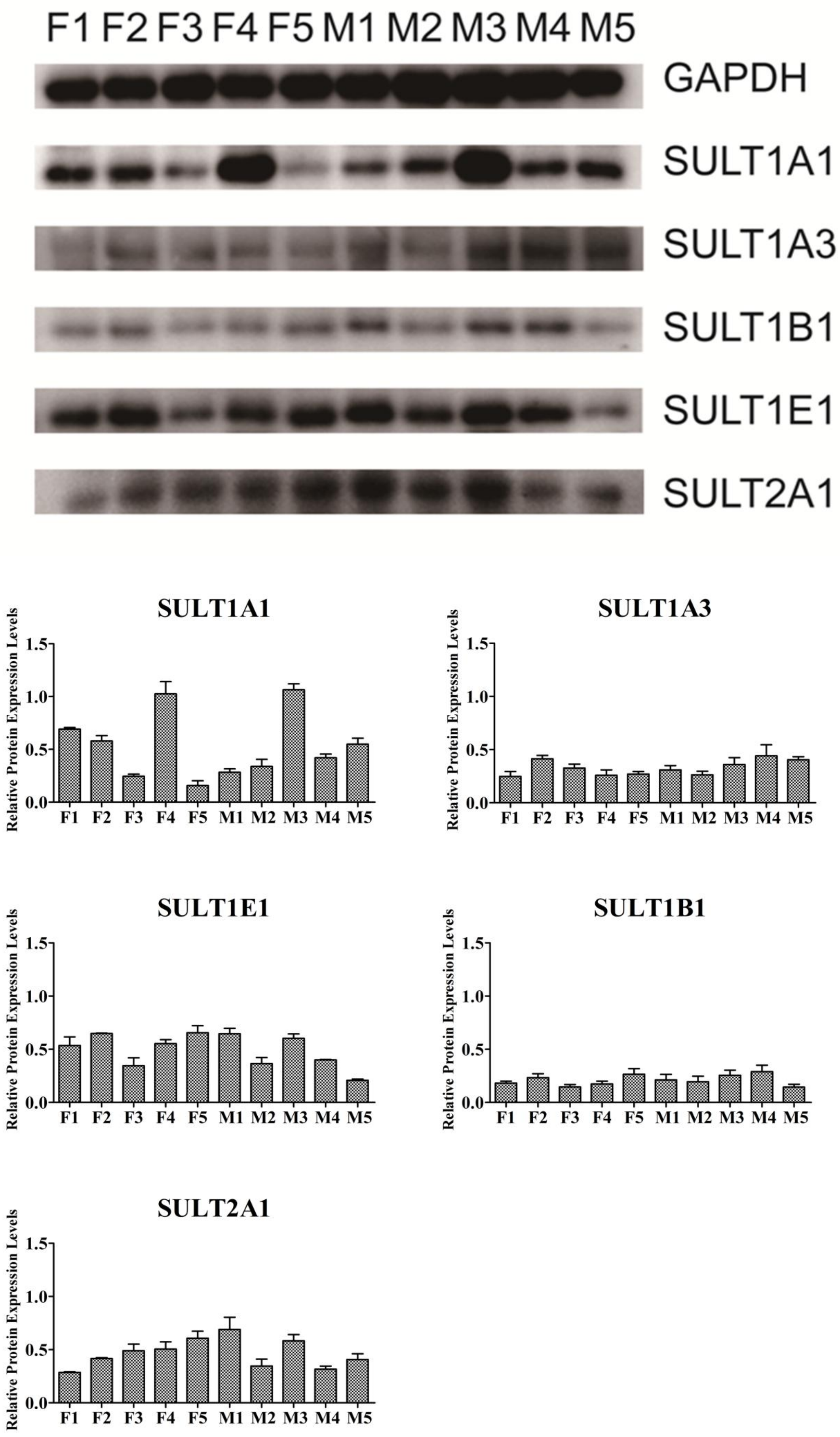
F1 F2 F3 F4 F5 M1 M2 M3 M4 M5
GAPDH
SULT1A1
SULT1A3
SULT1B1
SULT1E1
SULT2A1
SULT1A1
SULT1A3
SULT1E1
SULT1B1
SULT2A1
Relative Protein Expression Levels
1.5
1.0
0.5
0.0
F1 F2 F3 F4 F5 M1 M2 M3 M4 M5

Figure S5. We used the Western blot analysis to semi-quantify the SULTs enzymes such as the SULT1A1, SULT2A1, SULT1B1, SULT1E1 and SULT1A3 in healthy human liver S9 from 10 individual donors. M: male. F: female. GAPDH was used for normalization.

References

1 Roepstorff, P. & Fohlman, J. Proposal for a common nomenclature for sequence ions in mass spectra of peptides. *Biomedical mass spectrometry* **11**, 601, doi:10.1002/bms.1200111109 (1984).

2 Johnson, R. S., Martin, S. A., Biemann, K., Stults, J. T. & Watson, J. T. Novel fragmentation process of peptides by collision-induced decomposition in a tandem mass spectrometer: differentiation of leucine and isoleucine. *Analytical chemistry* **59**, 2621-2625 (1987).
